# The Immunogenicity in Mice of HCV Core Delivered as DNA Is Modulated by Its Capacity to Induce Oxidative Stress and Oxidative Stress Response

**DOI:** 10.3390/cells8030208

**Published:** 2019-02-28

**Authors:** Juris Jansons, Irina Sominskaya, Natalia Petrakova, Elizaveta S. Starodubova, Olga A. Smirnova, Ekaterina Alekseeva, Ruta Bruvere, Olesja Eliseeva, Dace Skrastina, Elena Kashuba, Marija Mihailova, Sergey N. Kochetkov, Alexander V. Ivanov, Maria G. Isaguliants

**Affiliations:** 1 Department of Pathology, Riga Stradins University, LV-1007 Riga, Latvia; juris.jansons@rsu.lv; 2 Latvian Biomedical Research and Study Centre, LV-1067 Riga, Latvia; Irina@biomed.lu.lv (I.S.); kate@biomed.lu.lv (E.A.); Bruvere@biomed.lu.lv (R.B.); daceskr@biomed.lu.lv (D.S.); mary@biomed.lu.lv (M.M.); 3 Department of Microbiology, Tumor and Cell Biology, Karolinska Institutet, SE-171 77 Stockholm, Sweden; elena.kashuba@ki.se; 4 N.F. Gamaleya Research Center of Epidemiology and Microbiology, Ministry of Health of the Russian Federation, 123098 Moscow, Russia; nvpetrakova@hotmail.com (N.P.); estarodubova@gmail.com (E.S.S.); olesenka80@mail.ru (O.E.); 5 Engelhardt Institute of Molecular Biology, Russian Academy of Sciences, 119991 Moscow, Russia; o.smirnova.imb@gmail.com (O.A.S.); kochet@eimb.ru (S.N.K.); aivanov@yandex.ru (A.V.I.); 6 RE Kavetsky Institite of Experimental Pathology, Oncology and Radiobiology, The National Academy of Sciences of Ukraine, 03022 Kyiv, Ukraine; 7 MP Chumakov Center for Research and Development of Immune and Biological Preparations of RAS, 108819 Moscow, Russia

**Keywords:** Hepatitis C virus, nucleocapsid (core), DNA-immunization, oxidative stress, cellular immune response, predictive marker

## Abstract

HCV core is an attractive HCV vaccine target, however, clinical or preclinical trials of core-based vaccines showed little success. We aimed to delineate what restricts its immunogenicity and improve immunogenic performance in mice. We designed plasmids encoding full-length HCV 1b core and its variants truncated after amino acids (aa) 60, 98, 152, 173, or up to aa 36 using virus-derived or synthetic polynucleotides (core191/60/98/152/173/36_191v or core152s DNA, respectively). We assessed their level of expression, route of degradation, ability to trigger the production of reactive oxygen species/ROS, and to activate the components of the Nrf2/ARE antioxidant defense pathway heme oxygenase 1/HO-1 and NAD(P)H: quinone oxidoreductase/Nqo-1. All core variants with the intact N-terminus induced production of ROS, and up-regulated expression of HO-1 and Nqo-1. The capacity of core variants to induce ROS and up-regulate HO-1 and Nqo-1 expression predetermined their immunogenicity in DNA-immunized BALB/c and C57BL/6 mice. The most immunogenic was core 152s, expressed at a modest level and inducing moderate oxidative stress and oxidative stress response. Thus, immunogenicity of HCV core is shaped by its ability to induce ROS and oxidative stress response. These considerations are important in understanding the mechanisms of viral suppression of cellular immune response and in HCV vaccine design.

## 1. Introduction

Hepatitis C virus (HCV) nucleocapsid (core) is the most conserved HCV gene [1,2,3]. Its quasispecies demonstrate unique homogeneity within the patients [4]. No genetic mutations have been detected in any of the CTL-epitope encoding core gene regions despite detectable cellular responses [1]. Altogether this indicates that it is subjected to very strong functional constraints. Conservation of HCV core and its vital role for the virus make it attractive as an HCV vaccine component. However, realization of this task turned to be quite difficult. Repeated attempts to induce potent anti-core immune response met with difficulties, even when using viral vectors [5,6,7,8]. HCV core was defined as a poor cellular immunogen, both when delivered as DNA and as a protein [9,10,11,12,13,14]. We have shown that plasmids directing higher levels of core expression were less immunogenic than directing lower levels of HCV core expression [5]. Several studies observed that transient expression of HCV core interferes with the induction of cellular immune responses against co-delivered genes [15,16]. In lines with these findings, mice transgenic for HCV core demonstrated significantly diminished T-lymphocyte responses compared to their non-transgenic littermates [17], and could not adequately respond to viral infections [18,19].

Poor immunogenicity of HCV core in experimental settings is likely attributed to its immunoregulatory properties. It interferes with numerous signaling pathways in the host cells, dysregulates promoters of various host genes and modulates apoptosis [20,21,22]. Specifically, it represses several interferon-stimulated genes, the targets of interferon regulatory factor 1 (IRF-1) such as interleukin-15 (IL-15) and IL-12 [23]. In pattern recognition receptor signaling, HCV core and its circulating forms suppress the production of type I and III IFNs in response to TLR and RIG-I agonists [24]. HCV core can also selectively suppress IL-2 promoter activity and decrease the production of IL-2 in response to T-cell receptor triggering, at the same time increasing the expression of tumor necrosis factor (TNF)-alpha, and Th2-type cytokines [25]. We, and others, have observed that HCV core mediates an increased production of transforming growth factor β1 (TGFβ1) [26,27]. The latter regulates cell proliferation, growth, differentiation, and exhibits strong immunomodulatory effects [28]. Specifically, TGFβ1 demonstrates a “manic-depressive behavior” [29] towards cells of the innate immune system, including NK cells, bridges mast cells and Tregs, and suppresses interferon (IFN)-γ production by CD4 T cells at both priming and recall [30]. Importantly, the immunomodulating/immunosuppressive effects are exerted not only on the expressing cells, but also the by-stander cells, which are affected by the circulating extracellular forms of the protein [24,31,32].

Through prohibition of the transcriptional factor binding to the IFN-stimulated response elements (ISRE), it down-regulates the transcription of interferon-induced antiviral genes, specifically of signal transducer and activator of transcription (STAT) 1 and STAT2 [33,34]. HCV core has also been implicated in the modulation of activation of the nuclear factor kappaB (NF-kappaB) family and manipulations with both apoptotic and antiapoptotic pathways [35]. The NF-kB/IkB family promotes the expression of over 100 targets, the majority of which participates in the host immune response [36]. This includes multitude of cytokines and chemokines, receptors required for immune recognition, proteins involved in antigen presentation, and adhesion receptors involved in transmigration across blood vessels walls. HCV core promotes activation of NF-κB [37] and makes cells more resistant to apoptosis induced by TNF-α [38]. Conversely, it may suppress activation of NF-κB by TNF-α, okadaic acid, phorbol ester, and hydrogen peroxide regulated cell death [35,39,40], acting on a step common to these NF-κB inducers [41]. These conflicting effects indicate that HCV core can induce aberrant effects on NF-κB signaling and, thus, contribute to the development of inflammatory and malignant disorders [42].

Nevertheless, HCV core has been incorporated into a number of clinical trials of HCV vaccines, alone (HCV Core ISCOMATRIX [9]) or as a component of a therapeutic DNA vaccine (CIGB-230; [43,44,45,46]). Studies revealed no toxic effects of immunization, the reported adverse events were generally mild to moderate in severity, of short duration and self-limiting [9,43]. Patients with chronic hepatitis C demonstrated encouraging results with respect to an improvement in liver histology [43]. Safety of HCV core preparations in the human trials paved the way to the continuous efforts to improve its immunogenic performance. Promising results were obtained with the chimeras of HCV core protein with HBV precore regions [47], tetanus toxoid [48], HCV core carried by Lambda phage nanoparticles [49] or an attenuated Salmonella strain [15], demonstrating the possibility to overcome core-driven immune suppression.

The arrival of highly effective and convenient treatment regimens promises the worldwide HCV eradication. However, resistance to these drugs is increasing [50], indicating the necessity to reinforce medication with immunotherapy which would limit viral replication and evolution towards resistant phenotypes. Today, sorafenib and regorafenib are the only therapeutic agents that have been demonstrated to have an effect in the advanced HCC [51]. However, the issue is controversial. DAA treatment were reported to prevent the recurrence of HCC after the remission [52], and, on contrary, to increase the recurrence of HCC and its aggressiveness [53] although some of these data, specifically for patients with cirrhosis, may be explained by patient characteristics and regularity of screening [54]. Even if DAA treatment is uniformly accepted to reduce the risk of acquiring liver cancer, it does not eliminate it completely [55]. Furthermore, HCC reduces the success rate of DAA treatment [56] and does not cure cancer unless the patient receives liver transplantation [57]. Clearly, novel curative approaches are urgently needed. The global burden of HCV infection, and concomitant liver cancer is unlikely to diminish unless there is a vaccine preventing chronic infection/chronic liver disease. HCV core would be the likely candidate provided it is devoid of its immunomodulatory/immunosuppressive properties.

Hereby, we attempted to link the molecular properties of HCV core when expressed in the eukaryotic cells to its performance in DNA immunization, identify the domains involved in immune suppression and define a way to design an optimized HCV core-based gene immunogen devoid of the immunosuppressive properties. We found that cellular immunogenicity of HCV core depends on its capacity to induce an oxidative stress and oxidative stress response, and that the immune suppression can be relieved by the deletion of the HCV core C-terminus harboring the domain involved in association of the protein with mitochondria, and generation of ROS attributable to the isoform 2E1 of cytochrome P450.

## 2. Materials and Methods

### 2.1. Plasmid DNA

The synthetic gene encoding amino acids (aa) 1–152 of HCV core has been described previously [58]. Plasmids carrying the synthetic polynucleotide encoding HCV core aa 1–152 (pCMVcore152s) and the full-length HCV core of HCV 1b strain 274933RU (Gene Bank accession #AF176573) under the control of the immediate early cytomegalovirus (pCMVcore191v) or the elongation factor 1-alpha promoters (pCMVcore191e) were described earlier [59,60]. HCV core coding sequence was cleaved from pCMVcore191v using the restriction endonucleases *Hind* III and *Xho* I and inserted into the eukaryotic expression vector pVax1 (Invitrogen, Carlsbad, CA, USA) under the control of the cytomegalovirus (CMV) immediate early (IE) promoter and polyadenylation signal from the bovine growth hormone gene generating plasmid pVaxCore191v. A TAGTAA sequence carrying two stop codons was inserted into one of the four sites of its coding sequence with the help of the kit for site-directed mutagenesis (Promega, Madison, WI, USA) to generate a panel of plasmids encoding HCV core proteins truncated after amino acids 60 (pCMVcore60v), 98 (pCMVcore98v), 152 (pCMVcore152v), and 173 (pCMVcore173v). The luciferase-coding plasmid pVaxLuc was kindly provided by Anna-Karin Maltais (Karolinska Institutet, Stockholm, Sweden). Plasmids were propagated in the *Escherichia coli* strain DH5alpha. Plasmid DNA was extracted and purified by Endo Free plasmid Maxi kit (Qiagen GmbH, Hilden, Germany). The purified plasmids were dissolved in the phosphate buffered saline (PBS) and used for in vitro expression assays and for DNA immunization.

### 2.2. Recombinant Proteins and Peptides

Proteins representing HCV core aa 1–60, 1–98, 1–152, 1–173 (GenBank accession #AJ132997; [61]) were expressed in *E. coli* and purified by chromatography using Ni-nitrilotriacetic acid (NTA) resin as was described earlier [62]. Purified proteins were dissolved in PBS. Protein purity according to the Coomassie blue staining of SDS-PAGE gels was 95%.

Peptides covering core amino acids (aa) 1–20, 13–33, 34–42, 34–56, 63–80, 76–90, 106–126, 129–145, 141–160, and 155–177 basing on HCV 1b isolate 274933RU (GenBank: AF176573), a negative control peptide TTAVPWNAS from gp41 of HIV-1, and a peptide representing the immunodominant CD8+ T cell epitope of luciferase GFQSMYTFV (Luc peptide; LucP) were purchased from GL Biochem Ltd. (now ChinaPeptides Co. Ltd.; Shanghai, China). Peptides were purified by HPLC to 70% purity. Structure was confirmed by matrix-assisted laser desorption/ionization mass-spectrometry. In cellular immunogenicity assays, the peptides were pooled 1:1 (*w*/*w*) to represent aa 1–56, or 1–80, 76–126, and 129–177.

### 2.3. Eukaryotic Expression and Stability of HCV Core Protein Variants

Eukaryotic expression of HCV core variants was tested in human hepatoma Huh7 cells, hamster kidney fibroblasts (BHK)-21, and human embryonic kidney (HEK) 293 cells. HCV core expression experiments were performed as was described earlier [63]. In brief, Huh7, BHK-21, and HEK 293 cells were cultured in Dulbecco’s modified Eagle’s medium (DMEM) with 2 mM glutamine, 50 U/mL penicillin and 50 µg/mL streptomycin supplemented with 10% fetal bovine serum (FBS; Invitrogen Corporation, Carlsbad, CA, USA). Cells were seeded in six-well plates at a density of 3 × 10^5^ cells/well into antibiotic-free DMEM with FCS and glutamine, grown to 90–95% confluence and transfected with 1 µg of HCV core encoding or control plasmids mixed with a transfecting agents Lipofectamine 2000, or Lipofectamine-LTX (both by Invitrogen), Turbofect, or ExGen500 (both by Fermentas, Vilnius, Lithuania). 

HCV core expression was analyzed 24, 36, and 48 h post transfection. Proteasome inhibitors MG132 (5–50 μM) and epoxomicin (0.1–5 μM) (both Sigma Aldrich Co., St Louis, MO, USA) were added to the medium 24 h post transfection. Cells were cultured for the additional 24 h, harvested at the given time-points, washed with phosphate-buffered saline (PBS) and lysed. BHK-21 and HEK 293 cells were lysed in the Laemmly buffer. Huh7 cells were lysed for 30 min on ice in a buffer containing 50 mM Tris-HCl, pH 7.5, 150 mM NaCl, 1% NP-40, 0.25% sodium deoxycholate, 1 mM PMSF, and 1× protease inhibitor cocktail.

Stability (half-life) of HCV core vaciants was assessed by cycloheximide-chase assay performed 24 h post transfection. Cells were treated with cycloheximide (Sigma-Aldrich , St. Louis, MO, USA) at the final concentration of 100 μg/mL. Cell aliquots were harvested immediately after and at hours 2, 4 and 24 post cycloheximide addition. Harvested cells were lysed, and the accumulation of HCV core variants was analyzed by 10 to 12 % SDS-PAGE and Western blotting as described above.

### 2.4. Western Blot Analysis

Cell lysates were analyzed by Western blotting as was described previously [63,64]. In brief, cell lysates were separated by SDS-PAGE using 10 to 12% gels, proteins were transferred in a buffer containing 25 mM Tris, 192 mM glycine and 20% methanol onto pre-blocked (1 h at room temperature with 5% nonfat milk in PBS) nitrocellulose Hybond-ECL or PVDF membranes (both from GE Healthcare Amersham, Waukesha, WI, USA). Membranes were incubated with primary rabbit anti-core serum (1:500) [59] at 4 °C overnight and then for 1 h at RT with the secondary HRP-conjugated goat anti-rabbit antibodies (DAKO, Glostrup, Denmark). In between incubations, membranes were washed twice for 10 min with PBS containing 0.5% Tween-20. Antibody binding was visualized using either ECL detection system (GE Healthcare Amersham, UK) or Pierce SuperSignal West Femto Kit (Thermo Scientific Pierce, Rockford, USA). After HCV core detection, blots were striped according to the detection system protocols and re-stained with mouse monoclonal anti-actin or anti-tubulin antibodies (Sigma-Aldrich, St. Louis, MO, USA) and HRP-conjugated anti-mouse antibodies (DAKO) for signal normalization. Immunoblots or ECL photos of immunoblots were scanned, and signals of the individual bands were quantified using the ImageJ software (http://rsb.info.nih.gov/ij). PAGE and Western blotting for the quantification of expression of HO-1 and Nqo-1 in Huh7 cells transfected with HCV core variants performed as was described previously [63].

### 2.5. Protein Stabilization Assays

Inhibition of proteasomal proteolysis in cell culture was performed by adding the proteasomal inhibitor MG132 (Calbiochem, San Diego, CA, USA) in concentration of 5 and 50 µM, and epoxomicin (Calbiochem) in concentration 50 µM. Inhibitors were added to the cell culture 24 h posttransfection. The cells were co-cultured with inhibitors for the additional 18 h, and then lysed and analyzed by immunoblotting.

### 2.6. Confocal Microscopy

Transfected cells were grown on the glass coverslips or chamber slides (Nunc, Roskilde, Denmark) for 24 to 48 h. Cell membranes were visualized by staining cells with 7 µg/mL of fluorescein isothiocyanate (FITC) conjugated Wheat germ agglutinin (WGA; Molecular Probes, Eugene, OR, USA) in PBS. After that, cells were fixed with 3,7–4% paraformaldehyde in PBS for 10 to 20 min. Fixed cells were permeabilized with 0.2% Triton X-100 in PBS for 3 min, blocked with PBS containing 2% of BSA and 0.2% Tween-20, and incubated first with anti-core rabbit serum diluted 25 to 250 in the Dako Cytomation Antibody Diluent (Dako Cytomation, Glostrup, Denmark) and then up to 1 h with FITC- or TRITC-conjugated goat anti-rabbit IgG (F9887 or T6778, respectively, Sigma). Finally, DAPI was added at 1 µg/mL for 5 min. All incubations were done at 20 °C. Slides were then mounted with Prolong Gold Antifade Reagent (Molecular Probes), covered with cover slips and read on a Leica TCS5 laser scanning confocal microscope (Leica, Wetzlar, Germany). Images were scanned and quantified using ImageJ software.

### 2.7. Measurement of Production of the Reactive Oxygen Species

Intracellular reactive oxygen species (ROS) production was measured by epifluorescence using the described protocol [26]. Briefly, the Huh7 cells were seeded into 48-well plates, transfected with plasmids encoding HCV core variants. Cell culture medium was removed 30 h post transfection, and cells were incubated in the medium containing 25 µM 2′,7′-dichlorodihydrofluorescein diacetate (DCFH-DA) for 30 min. The treated cells were washed with PBS, resuspended in PBS, and the fluorescence intensities were measured using Plate CHAMELEON V reader (Hidex Oy, Åbo, Finland) with excitation at 485 and emission at 535 nm.

### 2.8. Reverse Transcription with Quantitative PCR (RT-qPCR)

RNA was isolated from 5 × 10^5^ cells with PerfectPure RNA Cultured Cell kit (5Prime, Hamburg, Germany) and reverse transcribed using Mint Reverse transcriptase (Evrogen, Moscow, Russia) with random hexamer primer according to manufacturers’ protocol. Q-PCR was performed using IQ5 Real-Time PCR Detection System (Bio-Rad, Hercules, CA, USA) using the primers and the protocol reported previously [63]. Relative quantitative analysis was carried out by comparing threshold cycle number for the target genes and a reference β-actin mRNA, amplified in the separate tubes.

### 2.9. Animals

Experiments were carried in compliance with the bioethical principles adopted by the European Convention for the Protection of Vertebrate Animals Used for Experimental and Other Scientific Purposes (Strasbourg, 1986), Order of the Ministry of Health of the Russian Federation of 23 August 2010 “Establishment of the Rules of Laboratory Practice” No. 708n. Experiments were approved by the ethical committees of participating institutions: the Northern Stockholm Ethical Committee for the Animal Experiments (permit N66/13 16 May 2013), and Biomedical Ethics Committee of the Gamaleya National Research Center for Epidemiology and Microbiology, Ministry of Health of the Russian Federation (Protocol No. 10, 14 March 2017) and the Latvian Animal Protection Ethics Committee of the Latvian Food and Veterinary Service permission (permit Nr 99,1 June 2018. BALB/c and C57Bl/6 mice (females, 8–10 weeks old) were purchased from Charles River Laboratories (Sandhofer, Germany) or the Central Animal Breeding Facility “Krjukovo” of the Russian Medical Academy of Sciences, or Laboratory Animal Center, Institute of Biomedicine and Translational Medicine, University of Tartu, Tartu, Estonia. Mice were housed at a temperature of 22 °C under a 12 h light/dark cycle with ad libitum access to water and food. All animals were acclimatized for one week before starting the experiments. For invasive procedures such as intradermal and intramuscular injections and electroporation, mice were anesthetized by a mixture of 4% isofluorane with oxygen and maintained in 2.3% isofluorane flow administered through facial masks or by ether inhaled from the gauze-soaked nose cones.

### 2.10. Mouse Immunization Experiments

Immunization protocol 1 BALB/c mice were shaved, and injected in the skin to the left and to the right of the base of the tail with 10 μg of one of the following plasmids: pCMVcore191v (*n* = 7), pCMVcore191e (*n* = 4), pCMVcore173v (*n* = 4), pCMVcore152v (*n* = 4), pCMVcore152s (*n* = 6), pCMVcore98v (*n* = 3), pCMVcore60v (*n* = 3), or empty vector (*n* = 7), all dissolved in PBS. Plasmids were mixed 1:1 (*w*/*w*) with the reporter plasmid encoding firefly luciferase pVaxLuc. Immediately after the injections, the immunization sites were electroporated using DERMA VAX Clinical DNA vaccine delivery system (Cellectis Glen Burnie, MD). Electroporation was performed by placing a needle array electrode consisting of eight 2-mm pins arranged in two rows (1.5 × 4 mm gaps; BTX, #47-0040) over the skin and applying 2 pulses of 1125 V/cm delivered with a 50 μs interval, and 8 pulses of 275 V/cm delivered with a 10 ms interval. In a separate experiment, BALB/c mice were immunized by 25 μg of pCMVcore152s (*n* = 3) or empty vector (*n* = 3), each mixed with 25 μg of pVaxLuc, injected intramuscularly (i.m.) into the left and right hind legs. Plasmids were administered with in vivo transfection reagent Turbofect (Thermo Scientific, Waltham, MA, USA) according to the manufacturer instructions. Expression of Luc reporter was monitored 4, 11, 15, 22, and 26 days post immunization using the in vivo imaging technique (Spectrum, Perkin Elmer, Waltham, MA, USA). Mice were bled from the tail vein prior to and after the completion of immunization cycle. At the end of the experiment, mice were sacrificed, and spleens were collected.

Immunization protocol 2 Groups of C57BL/6 mice (*n* = 20 in each) were immunized by three intramuscular injections of 25 μg of pCMVcore152s, or pCMVcore191v, or empty vector, all dissolved in PBS, at weeks 1, 2, and 4. Mice were bled prior to, and 1.5–2 weeks after each immunization. At 1.5 and 2 weeks post prime, one and two weeks post boost 1, and two and six weeks post boost 2, three to four mice per group were sacrificed, and spleens were collected.

### 2.11. Preparation of Murine Splenocytes and Evaluation of Cytokine Secretion by Sandwich ELISA and IFN-γ/IL-2 Fluorospot Tests

The PBMCs from blood and splenocytes from spleens of immunized mice were isolated as described in [65]. The number of dead cells was below 5%. To assess proliferative immune responses, splenocytes were cultured for 1–4 days at 37 °C in 5% CO_2_ in the complete RPMI medium in the presence of HCV-derived and control antigens. T-cells were stimulated in triplicates with one of the following: Conconavalin A (ConA, 5 μg/ml; positive control), HCV core protein variants, or core derived peptides at 10 μg/ml. After three days incubation, 50 mcl cell culture fluids per well were removed, those from triplicate wells were pooled, and assessed for the presence of IFN-γ, IL-2 and IL-4 by Quantikine Sets (Pharmingen, San Diego, CA, USA). 

The number of IFN-γ and IL-2 producing cells was assessed by dual IFN-γ/IL-2 FluoroSpot assay (MabTech AB, Stockholm, Sweden) according to the manufacturer’s instructions. Splenocytes (2.5 × 10^5^ per well) were plated in the complete RPMI and stimulated with one of the following: Concanavalin A (ConA; 5 μg/mL) as positive control, medium alone, Luc peptide (10 μg/mL), recombinant HCV core aa 1–152 (5 μg/mL), or peptides representing core aa 1–20, 13–33, 34–42, 34–56, 63–80, 76–90, 106–126, 129–145, 141–160, and 155–177 either single or pooled in the equimolar amounts to the total concentration of 10 μg/mL, all in duplicate. Numbers of the spot-forming cells (SFCs) per million splenocytes were evaluated using an iSpot reader (AID GMbH, Strasberg, Germany; courtesy of Mabtech AB, Stockholm, Sweden).

### 2.12. Antibody ELISA

MaxiSorp ELISA plates (Nunc, Rochester, NY) were coated with the recombinant HCV core variants aa 1–98, or 1–152, or 1–173 (at 0.3 μg/mL) by incubating overnight at 6 °C or synthetic peptides representing HCV core (at 10 μg/mL) by incubating first for 20 h at 20 °C, and then for three days at 6 °C. All antigens were diluted in the carbonate buffer pH 9.3. ELISA was performed as described by us earlier [66]. Values of optical density (OD) of each well at 650 nm were subtracted from OD at 450 nm. 

ELISA performed on the plates coated with core aa 1–98, 1–152, and 1–173 demonstrated similar results. The average optical absorption values demonstrated by the sera of mice immunized with the empty vector at each of the dilutions, and standard deviation (SD) of each individual serum from the average were calculated. Cut-off values to discriminate sera containing core-specific antibodies from the negative sera were defined as an average OD_450–650_ of the control mouse sera at the given dilution plus 3 STDEV. All sera demonstrating at a given dilution an OD_450–650_ > the cut-off value were considered as antibody-positive. OD_450–650_ values of all serum samples and the cut-off values were plotted on a logarithmic scale to generate the titration curves. The end-point titer of specific antibodies was determined as the dilution factor at which the optical absorption of a sample crossed the cut-off curve.

### 2.13. In Vivo Imaging of Luciferase Gene Expression

In vivo imaging of the bioluminescence from the sites of the injection of the plasmid mixtures containing reporter plasmid encoding firefly luciferase (pVaxLuc) was performed with a highly sensitive CCD camera, mounted in a light-tight chamber (Spectrum, Perkin Elmer, Norwalk, CT, USA) as was described by us earlier [67]. To quantify the photon emission, a universal frame was selected which engulfed each of the individual photon-emitting areas cross groups and time-points. The frame was applied to all images in the series, and photons emitted from the given area per min were acquired. Data within one time-point were presented as an average of two sites in one mouse, and of all sites in the group of mice ±SD. Background luminescence was below 200 pixels/s/cm^2^.

### 2.14. Statistical Analysis

Statistical analysis of the in vitro data was performed with BioStat 2008 software (AnalystSoft, Vancouver, BC, Canada). All data was presented as means ± SD. Differences between two groups were compared using paired Student’s *t*-test. For comparison between multiple groups, ANOVA followed with Tukey–Kramer post-hoc test was applied. A value *p* < 0.05 was considered as statistically significant. In in vivo experiments, continuous but not normally distributed variables, such as the antibody levels, number of cytokine-producing spot-forming cells, or photon flux, were compared by the nonparametric Kruskal–Wallis and Mann–Whitney *U* tests. The Spearman rank-order correlation coefficient was calculated to characterize the linear correlations between variables. Calculations were done using STATISTICA AXA 10.0 (StatSoft Inc., Tulsa, OK, USA).

## 3. Results

### 3.1. Design of DNA Immunogens Encoding the Full-Length and Truncated Variants of HCV Core 

**Figure 1:**
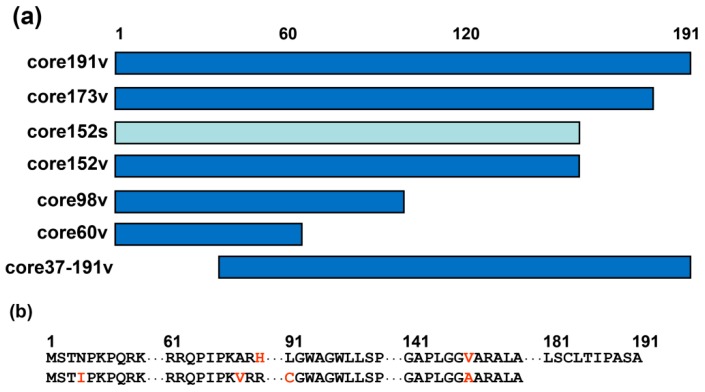
HCV core variants enrolled into DNA immunization. (**a**) Schematic representation of truncated proteins based on HCV core encoded by HCV genotype 1b strain 274933RU [71] (viral, v; in dark blue) or the synthetic DNA [58] (s, in light blue). Abbreviated names of the respective genes are given to the right. (**b**) Amino acid sequences of HCV core genotype 1b, strain 274933RU [68] (upper raw), and HCV core aa 1–152 encoded by the synthetic gene [58] (lower raw). Alignment of core191v and core152s showing the first and the last amino acid residues and regions of amino acid differences (in red).

Plasmids based on pVax1 carrying coding sequence of the full-length core of HCV genotype 1b strain 274933RU (core191v) and of its C-terminally truncated variants core60v, core98v, core152v, and core173v were inserted into pVax1 under the control of the immediate early CMV promoter and the bovine growth hormone (BGH) polyadenylation signal. The full-length HCV core was also expressed from the constitutive human elongation factor alpha promoter (EF1α; dubbed core191e) [60]. Graphical representation of HCV core variants is given in Figure 1. We took into the study also synthetic DNA encoding core of HCV genotype 1b strain HCV-J D90208 [68] (Figure 1). The synthetic gene was designed to minimize formation of secondary structure elements within the coding sequence that may decrease the expression levels [58]. An A-rich sequence was in-built downstream of the hairpin to prevent the involvement of the translation initiation zone in the local or distal secondary structures and thus promote better expression [58]. Folding of the “synthetic” HCV core mRNA (www.bioinfo.rpi.edu; [69]) predicted the disruption of the conserved stem-loop structures [70] and the formation of a hairpin with ATG exposed on its loop (data not shown).

### 3.2. Eukaryotic Expression of HCV Core Variants

**Figure 2:**
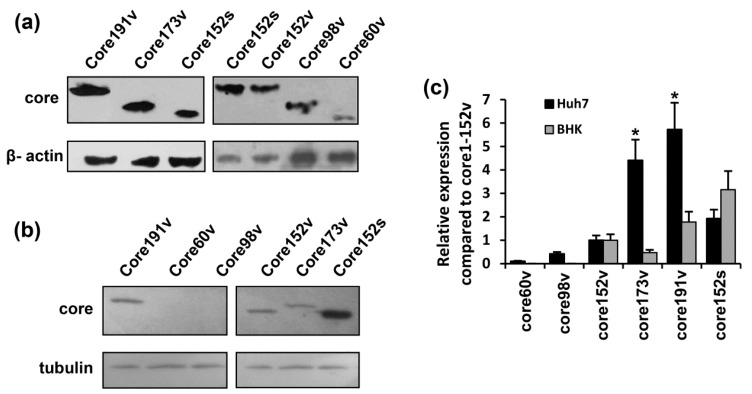
Eukaryotic expression of the full-length and truncated variants of HCV core protein. HCV core coding plasmids were used to transfect Huh-7 (**a**) or BHK-21 cells (**b**). In brief, 48 h post transfection, the cells were lysed, resolved by SDS-PAGE and subjected to Western blotting with anti-core rabbit antibodies [60] (**a**,**b**, upper panels). After core-specific staining, the blots were stripped and re-stained with antibodies to tubulin or β-actin (**a**,**b**, lower panels). (**c**) The signals generated in each experiment were quantified using ImageJ, core-specific signals were normalized to that of actin for Huh7, or tubulin for BHK-21 cells, and presented as fold-difference to the level of expression of core151v (altogether four separate experiments). Core173v and 191v were better expressed in Huh7 cells (* *p* < 0.05); levels of expression of core152s in these two cell lines did not differ.

Eukaryotic expression of HCV core variants in BHK-21, Huh7, and HEK293 cells was confirmed by Western blotting and by the immunofluorescent staining (Figure 2, and Supplementary Figure S1, data not shown). The level of expression of HCV core variants decreased in tact with the C-terminal truncation (Figure 2a–c). Huh7 cells transfected with core191v gene produced approximately 100 fg per cell of the core protein [63]. The level in the cells of the shorter versions encoded by core173v and core152v could then be estimated as 80 and 20 fg per cell, respectively (Figure 2c). The shortest form core aa 1–60 was expressed at up to 100 times lower level than the parental full-length protein and was barely detectable (for expression in Huh7 cells, see Figure 2a,c; for expression in BHK-21 cells, see the section on protein stability and processing). The N-terminally truncated core 37–191 could only be visualized by the immunofluorescence staining (Supplementary Figure S1a). Devoid of the immunodominant epitope on the N-terminus, this form could be recognized by only a fraction of rabbit antibodies directed against the subdominant epitope(s) residing in the central region of the protein [72]. Expression from the synthetic gene provided up-to 40 fg of HCV core152 per cell (Figure 2c). Core152s demonstrated a more uniform expression across the cell lines tested (Figure 2c).

HCV core aa 1–191 and aa 1–173 resided predominantly in the cytoplasm as demonstrated by the immunofluorescence staining of the expressing cells and confirmed by the laser scanning confocal microscopy (Supplementary Figures S1a,b and S2). Cells transfected with core191v and core173v DNA exhibited a granular staining pattern typical for the protein localization in the endoplasmic reticulum (ER). HCV core aa 37–191 was localized in the cytoplasm/perinuclear space as the full-length protein (Supplementary Figure S1). HCV core variants encoded by core152v and core152s DNA were mainly localized in the nucleus (Supplementary Figure S1), in lines with the earlier observations [73,74,75,76]. Confocal microscopy confirmed the translocation of HCV core aa 1–152 into the nucleus (see Figure S2a, panel III for typical staining). Ratio between the nuclear and cytoplasmic fractions (N/C) was equal to 0.5 for core191; 1.1 for core173; and 1.24 for core152, respectively (Supplementary Figure S2c). We calculated also the ratio R between the nuclear and the cytoplasmic forms according to Leclerc et al. (R = (N − C)/(N + C); [77]). For core191, R was negative (−0.38; Supplementary Figure S2c) supporting its cytoplasmic localization (Supplementary Figure S2aI,bI). For core172, it was positive, but quite low (0.05; Supplementary Figure S2c) suggesting that a small proportion of the protein was localized in the nucleus (Supplementary Figure S2aII, bII). Low N/C and R values for core152 (0.11; Supplementary Figure S2c) confirmed its nuclear localization (Supplementary Figure S2aIII, bIII).

### 3.3. Proteasomal Degradation of HCV Core Variants in BHK-21 Cells

The stability and degradation of HCV core variants was tested in the BHK-21 cells. Pulse-chase experiments with cycloheximide demonstrated that HCV core aa 1-191, 1-173 and 1-152 had similar half-lives of approximately 10 to 12 h (Supplementary Figure S3), while half-life of the shorter core variants was reduced to less than two hours (data not shown).

**Figure 3:**
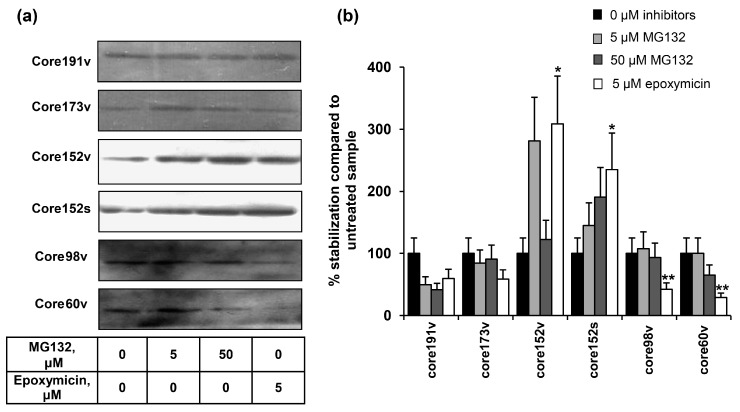
The effect of proteosome inhibitors on the accumulation of HCV core protein variants. (**a**) Western blotting with anti-core rabbit antibodies after transfection of BHK-21 cells and 24 h incubation (lane 1), and additional 24 h incubation with proteosome inhibitors with subsequent lysis (lanes 2–4); (**b**) Quantification of images using ImageJ software; signals from the cell cultures treated with proteasome inhibitors were normalized to the signal generated by respective HCV core variant in untreated cells; Y axis shows % stabilization. Data represent the results of two independent experiments. * Core1-152 was better stabilized by epoxomycin treatment than other HCV core forms (*p* < 0.05); ** core1-98 and core1-60 were not stabilized by epoxomycin treatment as compared to all other HCV core forms (*p* < 0.05; Tukey–Kramer test).

Proteasome-specific degradation of the truncated HCV core variants was tested by treating BHK-21 cells transfected with HCV core gene variants with the specific inhibitors, MG132 or epoxomicin. Epoxomycin is an irreversible proteasome inhibitor with a high specificity for a small class of the N-terminal nucleophilic hydrolases constituting 20S proteasome core particle and does not inhibit other proteases (as calpain, trypsin, chymotrypsin, papain, or cathepsins) [78]. MG132 is a transition-state inhibitor of primarily the chymotrypsin-like activity of 26S proteasome, but can also affect some of the lysosomal cysteine proteases and calpains [79]. We have not detected any effect of either MG132, or epoxomycin on the full-length core191 (it was degraded also in their presence; Figure 3). Proteasomal inhibitors prevented the degradation of core173, without supporting its accumulation (Figure 3). Core152 demonstrated a completely different pattern. It was strongly stabilized by both MG132 and epoxymicin (Figure 3). Stabilization by epoxomycin was significantly stronger that for other core forms (Figure 3b), indicating preferential processing of core152 by the proteasome. Shorter core variants were stabilized by MG132, but not epoxomycin, indicating a non-proteasomal degradation (Figure 3). 

### 3.4. Both the Full-Length and the C-Terminally Truncated Versions of HCV Core Induce the Production of Reactive Oxygen Species (ROS) and an Oxidative Stress Response

**Figure 4:**
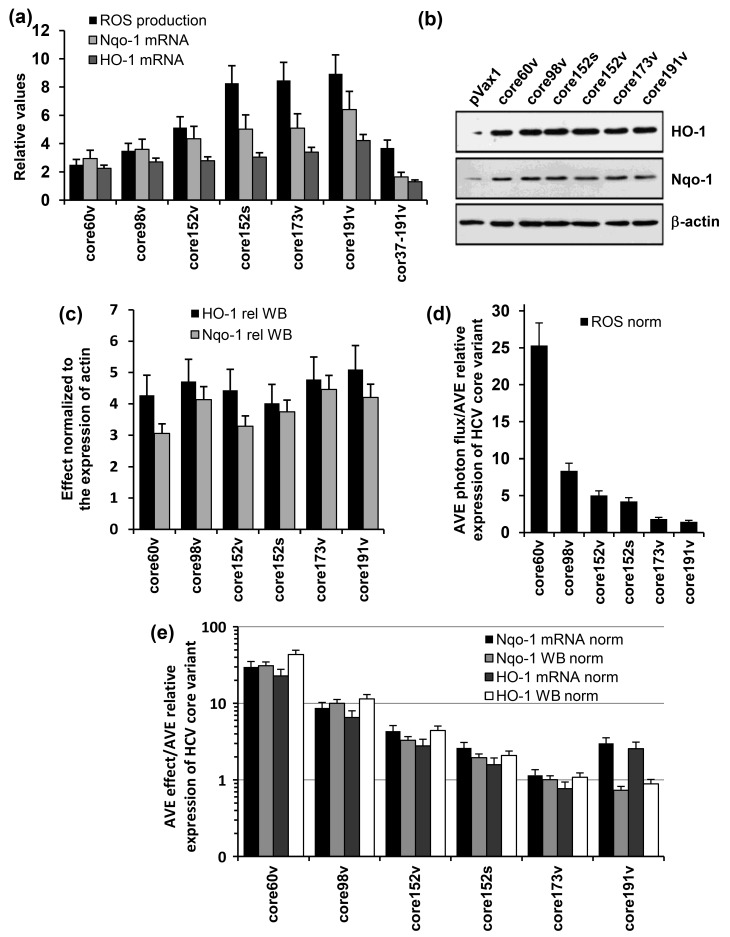
The induction of oxidative stress and oxidative stress response by variants of HCV core genes. (**a**) HEK293 cells were transiently transfected with plasmids core1-60v, core1-98v, core1-152v, core1-191v, core37-191v DNA based on the viral gene sequence, or core152s DNA based on the synthetic core1-152 encoding DNA, or empty vector pVax1. After 30 h, cells were assessed for the production of ROS with an oxidation sensitive dye dichlorofluoresceine diacetate (DCFHDA), and after 48 h, for activation of transcription of Phase II detoxifying enzymes Nqo1 and HO-1; (**b**) expression HO-1 (upper panel), Nqo1 (mid panel) as assessed by Western blotting in comparison to β-actin (lower panel) in cells expressing core variants; (**c**) expression of HO-1 and Nqo1 proteins quantified by ImageJ and normalized to the expression of β-actin; (**d**,**e**) the effects of HCV core variants on the production of ROS (**d**) and transcription and translation of HO-1 and Nqo 1 genes (**e**) normalized to the average level of expression of the respective protein variant represented as fold-expression of core1-152v (calculated for the samples analyzed in one experiment). Production of ROS was registered by microplate fluorimetry after treating cells with an oxidation sensitive dye dichlorofluoresceine diacetate (DCFHDA). HO-1 and Nqo 1 mRNA levels were quantified by RT-qPCR. Levels of HO-1 and Nqo1 enzymes were assessed by Western blotting. Total ROS flux and HO-1 and Nqo-1 mRNA levels (**a**) and expression of HO-1 and Nqo1 (**c**) were normalized to the respective levels exhibited by HEK293 cells transfected with the empty vector. Data represent the results of the duplicate runs with % deviation.

HCV core variants were tested for the capacity to induce an oxidative stress. For this, Huh7 cells were transfected with HCV core DNA and 30 h later (optimal timing in lines with our previous observations [63]) treated with the oxidation-sensitive dye 2′,7′-dichlorodihydrofluoresceine diacetate (DCFH-DA). They all induced the production of ROS manifested by an increase in the fluorescence registered by microplate fluorimetry (Figure 4a). ROS production was abrogated by pretreatment of cells with a ROS scavenger pyrrolidine dithiocarbamate (data not shown).

Next, we addressed the ability of HCV core variants to regulate the antioxidant defense pathway. Namely, we assessed their effects on the transcription of two phase II detoxifying enzymes: human NAD(P)H:quinone oxidoreductase (Nqo-1) and heme oxygenase 1 (HO-1). For this, we transfected Huh7 cells with HCV core DNA, or an empty vector, and 24 to 40 h post transfection assessed the levels of transcription and translation of Nqo-1 and HO-1 genes. HCV core variants with the intact N-terminus up-regulated the transcription and translation of both HO-1 and Nqo-1 (Figure 4a–c). Expression of the shortest one, core60v, enhanced the transcription of Nqo-1 and HO-1 from 2 to 3, and of the full-length core191, 4–6 times (compared to the levels of transcription of Nqo-1 and HO-1 in cells transfected with the empty vector; Figure 4a). Core152, core173 or core191 did not differ in their capacity to induce ROS, or up-regulate the transcription and translation of HO-1 and Nqo-1 genes (*p* > 0.05; Tukey–Kramer test). Intracellular content of both enzymes increased 3–5 times compared to that in the vector-transfected cells (Figure 4b,c). Deletion of the N-terminal domain of HCV core had deleterious effects both on the induction of ROS, and on the transcription of Nqo-1 and HO-1 (Figure 4a).

Keeping in mind that HCV core variants are expressed at different levels (Figure 2), we related the magnitude of ROS production and the extent of up-regulation of Nqo-1 and HO-1 to the amount of the variants typically detected in cell lysates at the time of measurement (an average value from the repeated experiments). The strongest (per protein unit) effect on the production of ROS and induction of the phase II detoxifying enzymes was generated by the heavily truncated core60 followed by core98 (Figure 4d,e). Truncations of the C-terminus up to aa 152 reduced the normalized levels of ROS production but had little effect on the normalized parameters of oxidative stress response, with concordant results of transcription and translation assays (Figure 4e). An increase of the protein content per cell from 40 (core152s) to 100 fg/cell (core191v) did not result in a proportional increase in the levels of ROS, or in the expression of Nqo-1 or HO-1. Relative effect of the N-terminal truncation could not be evaluated due to our inability to quantify the expression of core37–191 by Western blotting (as was done for the other core variants).

### 3.5. Immunogenic Performance of the Genes Encoding HCV Core Variants in BALB/c Mice 

**Figure 5:**
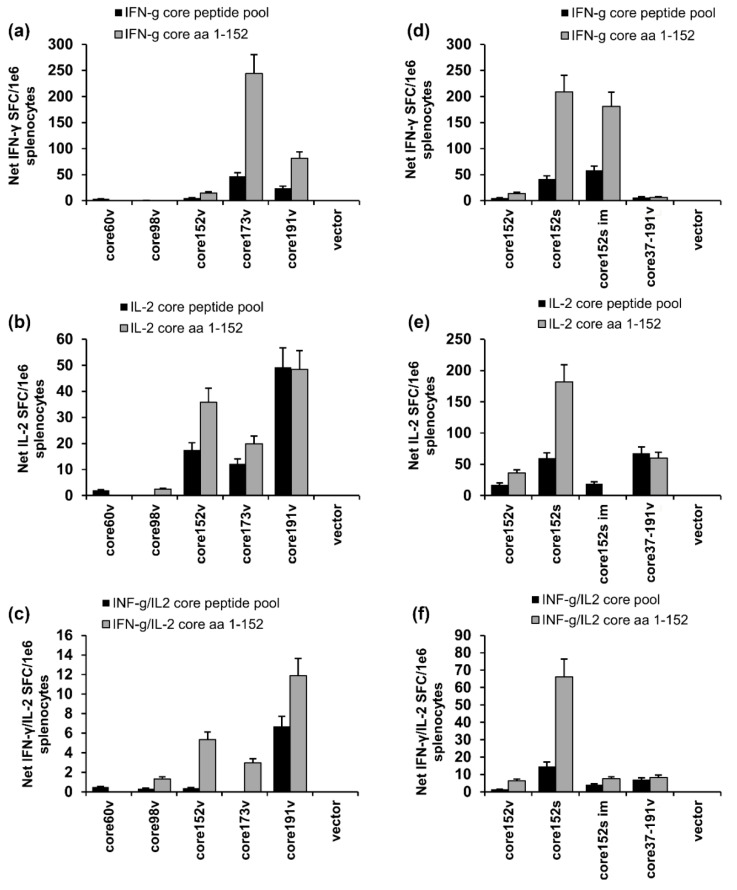
Cellular immune response of BALB/C mice to DNA-immunization with HCV core gene variants delivered by intradermal injections followed by electroporation. Comparison of the results of independent intradermal DNA-immunization/EP with core60v, core98v, core152v, core191v, core37-191v, and empty vector (**a**–**c**); or core37-191v, core152 encoded by DNA of viral (core152v) versus synthetic origin (core152s), also delivered by im injection without EP (core152s im) and empty vector DNA (**d**,**e**). Graphs show cytokine secretion of mouse splenocytes in response to in vitro stimulation with core-derived peptide pool and recombinant core152 represented as the number of spot forming cells per mln splenocytes producing IFN-γ (A, D); IL-2 (**b**,**e**); and both cytokines (**c**,**f**) assessed by IFN-g/IL-2 Fluorospot. All assays were done in duplicates. Results represent average values for all mice in the group ± SD. The specific responses exceeded three-times the secretion of respective cytokines in response to stimulation with an unrelated control peptide. No difference between the groups was registered in response to stimulation with the mitogen ConA (*p* > 0.05; data not shown).

Next, we attempted to relate the in vitro properties of HCV core variants to their performance in DNA immunization. Groups of BALB/c mice were intradermally (ID) injected with core1-60v, core98v, core152v, core152s, core37-191v, and core191v DNA, all controlled by CMV promoter, or core191v controlled by the human elongation factor 1a (EF-1α) promoter (core191e). The injections were followed by electroporation (EP). The immune response was assessed three weeks post immunization by dual IFN-γ/IL-2 Fluorospot and antibody ELISA. The highest specific IFN-γ response was registered in splenocytes of mice DNA-immunized with core173v, followed by core191v gene (Figure 5a). Core191v DNA induced also a weak IL-2 response (Figure 5b). Core191e DNA (controlled by EF1α promoter yielding 6–7 times less protein than CMV [60]) induced specific production of IFN-γ (*p* < 0.05), but no IL-2 (Supplementary Figure S4). Mice DNA-immunized with the N-terminally truncated core37-191v gene mounted a weak IL-2 response, as the recipients of core191v DNA (*p* > 0.1; Figure 5e). None of the virus-based DNA-immunogens was able to induce dual IFN-γ/IL-2 response.

We have further compared the performance in DNA immunization core152 encoded by viral and synthetic DNA (core151v and core152s; Figure 5d–f). Core152s DNA delivered by ID+EP regimen was superior in the capacity to induce IFN-γ (*p* < 0.05; Figure 5d). It also induced IL-2 and a dual IFN-γ/Il-2 response (Figure 5e,f). In a separate experiment, we delivered core152s gene by intramuscular injections (2 × 25 αg) without EP. This immunization induced an IFN-γ response comparable to that in the ID + EP regimen, but no IL-2 (Figure 5d–f).

In mice DNA-immunized with core173v and core191v antibody response was weak; truncation of core beyond aa 173 made it more variable (*p* > 0.05; Supplementary Figure S5). On contrary to that, all mice immunized with core152s DNA developed an antibody response in titers 4000 ± 500, significantly exceeding that in counterparts receiving core152v (*p* < 0.05; Supplementary Figure S5a). Intramuscular injections of core152s DNA without EP induced no humoral response (Supplementary Figure S5a).

### 3.6. The Immunogenicity of HCV Core Gene Variants Does Not Depend on the Mouse Strain or Immunization Route

We have further tested if our observations on the immunogenicity of HCV core gene variants would hold true in different settings, in another mouse strain and with a different mode of DNA delivery. For this, we DNA-immunized C57BL/6 mice with two representative core variants: more immunogenic core152s and less immunogenic core191v. Immunization was done by repeated intramuscular injections of respective plasmids without EP, to rule out unspecific immune activation due to EP-induced trauma and inflammation [80]. T-cell response to stimulation with HCV core and core-derived peptides was assessed by T-cell proliferation and registered as the stimulation indexes (SI) and by the levels of IFN-γ and IL-2 secretion done 1.5 and two weeks after the prime, two weeks after the first, and two and six weeks after the second boosts. We choose sandwich ELISA to detect production of cytokines, especially IL-2, since we have recently shown that binding of IL-2 in dual IFN-γ/IL-2 Fluorospot tests can deprive T-cells of the adequate stimulation and, thus, compromise cellular responses, specifically of CD8 + T cells [81].

**Figure 6:**
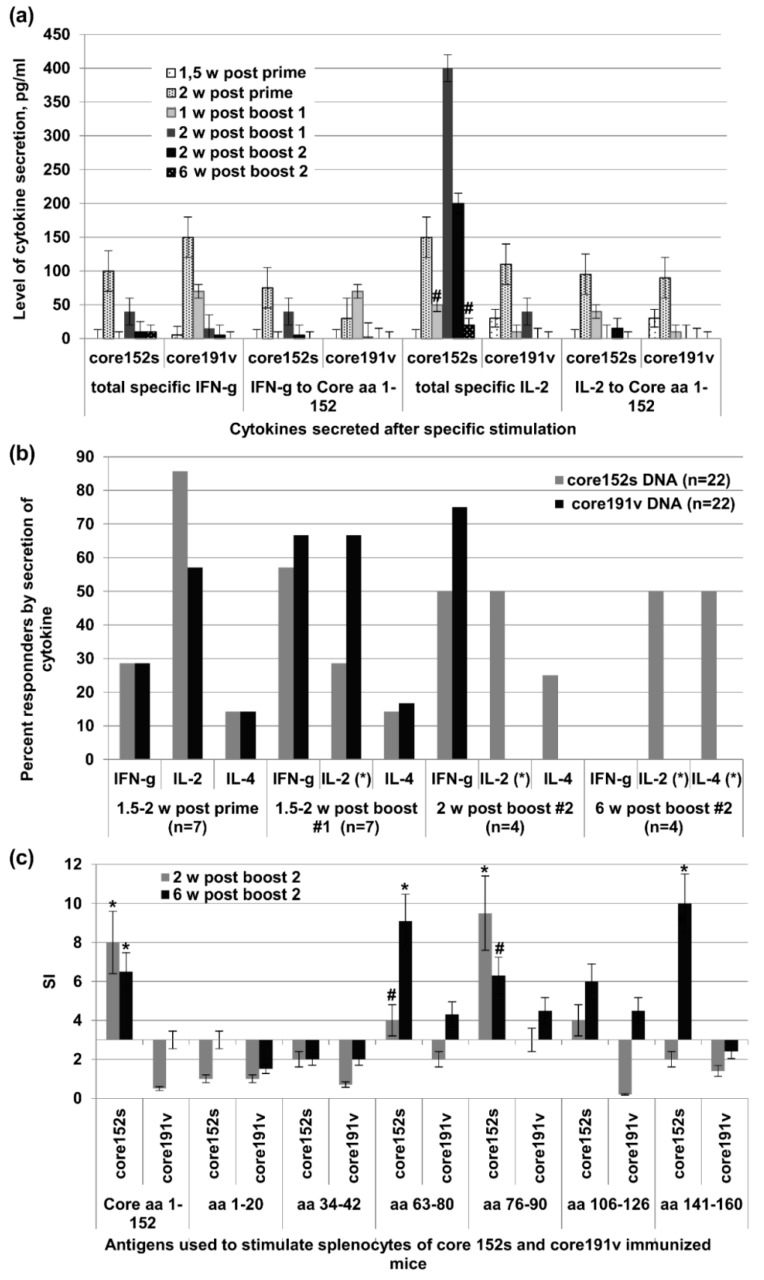
Cellular immune response of C57Bl/6 mice to DNA-immunization with HCV core gene variants delivered by intramuscular injections. (**a**) Secretion of IFN-γ and IL-2 by the splenocytes of mice DNA-immunized with core152s (*n* = 22) or core191v (*n* = 22) and stimulated with the recombinant HCV core or core-derived peptides 1.5 or two weeks after the prime, one and two weeks post the 1^st^ boost, and two or six weeks post the 2nd boost; (**b**) Percent of mice responding to T-cell stimulation by the secretion of IFN-γ, IL-2 and IL-4 at different time points of immunization cycle; (**c**) Proliferative response of mouse splenocytes to in vitro stimulation with HCV core and core-derived peptides two and six weeks post the last DNA immunization. * denotes a significant difference between the values demonstrated by the recipients of core152s and core191v DNA (*p* < 0.05); #-tendency for difference between the values demonstrated by the recipients of core152s and core191v DNA (0.05 < *p* < 0.15). Cellular response data for each time point correspond to a different set of mice sacrificed to perform the cellular test. All assays were done in duplicates. Statistical analysis was done by Mann–Whitney test.

After DNA prime, the immunogenicity profile of core152s and core191v did not differ (Figure 6a,b). In the test done 1.5 weeks post the 1st boost, recipients of core191v DNA recipients demonstrated higher levels of IFN-γ production in response to stimulation with core152 and core-derived peptides (*p* < 0.05), while core152s DNA recipients tended to mount a stronger IL-2 response (*p* = 0.11; Figure 6a). Two weeks post the 1^st^ boost, more mice in the core191v DNA-immunized group responded by IFN-γ and IL-2 secretion, but recipients of core152s DNA exhibited stronger core-specific secretion of IL-2 (*p* < 0.05), and a tendency to stronger IFN-γ response (*p* = 0.11; Figure 6a). These differences in immunogenicity increased after the 2nd boost. Recipients of core152s DNA demonstrated a strong specific IL-2 response, mostly to stimulation with HCV core derived peptides (*p* < 0.05; Figure 6a). Low level of secretion of IL-2 was observed in 50% of the animals of this group also six weeks post the 2nd boost indicating the formation of HCV core-specific memory response (Figure 6a,b). No such response was seen in mice boosted with core191s DNA (*p* = 0.1, *t* test; Figure 6a,b). Interestingly, we detected also a low level IL-4 response (5–10 pg/mL). It was observed in a minority of core152s and core191v DNA immunized mice after prime and 1st boost (Figure 6b). After 2nd boost, IL-4 production was detected in 50% recipients of core152s, but not core191v DNA (*p* = 0.1; Figure 6b).

We examined the specificity of cellular response by evaluating the capacity of mouse splenocytes to proliferate when stimulated with the individual HCV core-derived peptides. Both two and six weeks post the last immunization, splenocytes of core152s (but not of core191v) DNA recipients strongly proliferated in response to stimulation with the recombinant HCV core, and the peptides representing major T-cell epitopes of HCV core recognized in mice [82] at aa 63–80, 76–90, 106–126, and 141–160 (Figure 6c). Thus, by week 6 post the last immunization, mice receiving core152s gene had established a memory response to a vast array of the known T-cell epitopes of HCV core.

Antibody response induced by core191 gene was low and short-lasting, seroconversion was registered in 40% (8/20) animals with the average end-point titer of anti-HCV core antibodies 1:100 (Supplementary Figure S5b). On contrary, immunization with core152s gene led to 80% seroconversion (16/20; *p* = 0.01) with the average titer of 500 ± 100 (Supplementary Figure S5b). Anti-core antibodies induced by core152s DNA pertained long after the completion of the immunization cycle with the end-point titer of 1:300 six weeks post the 2nd boost (Supplementary Figure S5b).

### 3.7. In Vivo Assessment of Effector Immune Response

**Figure 7:**
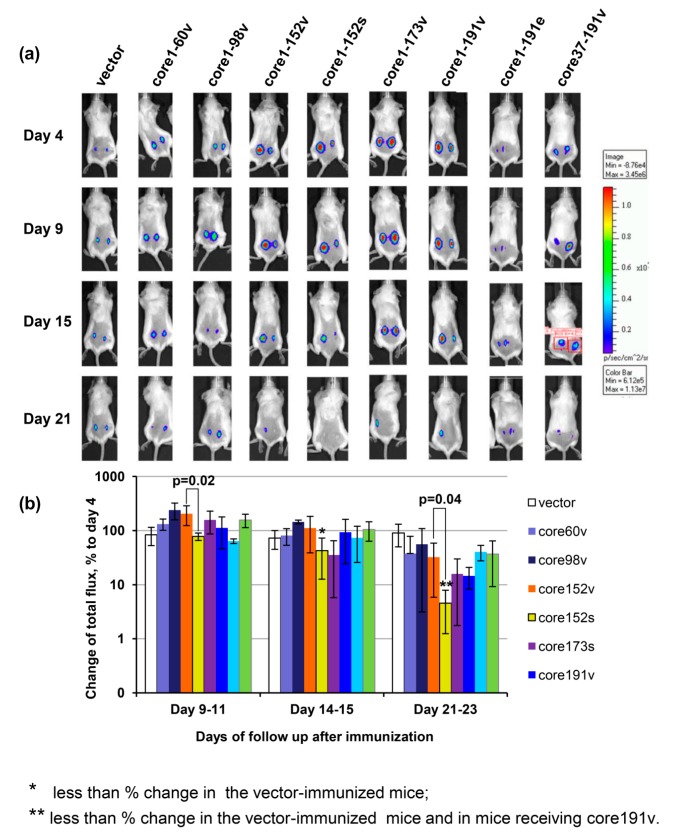
The effector potential of anti-core immune response in BALB/c mice visualized as the capacity to reduce bioluminescent signal emitted from the areas of co-delivery of HCV core variant and luciferase reporter encoding DNA. Composite image of representative mice receiving injections of plasmid encoding one of the HCV core gene variants mixed with plasmid encoding firefly luciferase (Luc; pVaxluc), showing bioluminescence from the injection areas to the left and to the right of the back, on days 4, 9, 15, and 21 post immunization. (**a**); Average luminescence from all injection sites in a group normalized to the level reached in this group by day 4 post immunization, in % ± SD (**b**); Correlations of IFN-γ and dual IFN-γ/Il-2 response to HCV core aa 1–56 with the loss of luminescence (Spearman rank test; *p* < 0.05) (**C**). Mice were immunized by intradermal injection of plasmids carrying viral (HCV 1b 274933RU) sequences for HCV Core aa 1–60 (60v), 1–98 (98v), 1–152 (152v), 1–173 (173v), 1–191 (191v), and 37–191 (37–191v) controlled by the CMV promoter, and core191v controlled by the EF1-a promoter (191e), or by the CMV-controlled synthetic gene for core aa 1–152 (152s) [50]. Control mice received pVax1. Plasmids were injected intradermally as 1:1 mixture with pVaxLuc. Injections were followed by electroporation. On days 4, 9 to 11, 14 to 15, and 20 and 22 post immunization mice were assessed for the total photon flux emitted from the areas of immunization. Emission was registered by a CCD camera in-built into the Spectrum imager (Perkin Elmer). Bar on the right side shows the color scale reflexing the intensity of photon flux (photons/sec/cm2/sr). Red squires on one of the mouse images depict a typical area with quantification of photon emission. * significant difference from the values exhibited by mice receiving empty vector; ** significant difference from the values exhibited by the control mice receiving empty vector and by the mice receiving core191v (*p* < 0.05; Mann–Whitney test). Difference in the total photon flux, and % change of the flux between mice receiving viral and synthetic genes for core152 are depicted over the respective bars (Mann–Whitney test).

The integral immune response against HCV core expressing cells was assessed by in vivo imaging. The latter allows to visualize the elimination of the expressing cells seen by monitoring the loss of bioluminescence from the sites of co-delivery of the target and reporter genes such as the firefly luciferase [81,83,84]. Mice immunized with core152s gene demonstrated a significant decrease in the levels of bioluminescence from the sites of co-delivery of HCV core/luciferase genes (Figure 7). Starting from day 9, the bioluminescent signal in the recipients of core152s DNA was significantly lower than in the control mice (*p* < 0.05; Figure 7b). After day 21, it was also lower than the bioluminescent signal registered in mice receiving core152v, or core191v DNA (*p* < 0.05; Figure 7b). Thus, an efficient immune response capable of efficient clearance of HCV core/reporter expressing cells from the sites of immunization was induced only by DNA immunization with synthetic gene encoding HCV core aa 1–152. Luminescence from the HCV core/reporter gene injection sites showed a weak inverse correlation to the magnitude of IFN-γ and dual IFN-γ/IL-2 response of mouse splenocytes to in vitro stimulation with a peptide pool covering aa 1–56 (R = −0.36; *p* = 0.01).

These experiments demonstrated that only DNA immunization with a synthetic gene encoding core aa 1–152 was able to trigger a long-lasting specific cellular response characterized by proliferation of lymphocytes with secretion of cytokines (INF-γ, IL-2, and/or IL-4) to multiple epitopes in HCV core, and production of specific antibodies, capable to clear cells co-expressing the reporter and the immunogen from the sites of immunization.

### 3.8. Immunogenicity of HCV Core Gene Variants in Mice Is Associated with Their Ability to Induce Oxidative Stress and Oxidative Stress Response

We tried to underpin the molecular correlates for such performance, i.e., an interdependence between the production of HCV core variants, induction of oxidative stress and molecular events of oxidative stress related response (dubbed “oxidative stress response”) and characteristics of the immune response. Namely, for each HCV core variant, we correlated the level of expression; its stabilization by proteasomal inhibitors; its capacity to generation of ROS, and induce the transcription and translation of Phase II detoxicating enzymes to the average number of splenocytes (per mln) producing INF-γ and/or IL-2 exhibited by BALB/C mice DNA immunized with this variant after in vitro stimulation with the recombinant protein, and pools of peptides covering aa 1–80, 76–126, and 129–177 (by Fluorospot).

No correlation was observed between Fluorospot results and the levels of eukaryotic expression of HCV core variants, or the degree of their stabilization by MG132 (*p* > 0.1; Spearman Rank Order test). A tendency was observed for the correlation of Fluorospot results with the degree of stabilization of HCV core variants by epoxomycin (R = 0.73, *p* = 0.09 for the total IFN-γ spots after stimulation with core-derived peptides; data not shown). Importantly, we identified a strong correlation of IFN-γ response to the ability of core variants to generate ROS and up-regulate the transcription of HO-1 (*p* < 0.05; Table 1). Correlations of IFN-γ production in response to peptides representing the N-terminal and central domains of HCV core protein became stronger when the levels of HO-1 transcription were normalized to the level of HO-1 transcription induced by pVax1 vector, and the levels of translation, to the actin content of the probes (R = 0.88 to 0.98; *p* < 0.05; Table 1). Same correlations were observed for the transcription and translation of Nqo-1 (data not shown).

We have also correlated the specific production of IFN-γ to the relative levels of ROS and HO-1 and Nqo-1 induced by each of HCV core variants per unit of this variant in a cell (Table 1). The amount of IFN-γ generated by HCV core variants, specifically in response to the epitopes in the N-terminal region of the protein, demonstrated a strong inverse correlation to the relative normalized levels of HO-1 and Nqo-1 (R = −0.88, *p* < 0.05; Table 1). No correlations were found between the levels of ROS, or HO-1, or Nqo-1, and the production of IL-2 or IFN-γ/IL-2 (data not shown). Thus, the capacity of HCV core variants to induce IFN-γ (but not IL-2 or IFN-γ/IL-2) response correlated with their capacity to induce ROS and trigger expression of HO-1 and Nqo-1.

## 4. Discussion

In this study, we aimed to reach a better understanding of the molecular mechanisms behind the immunogenic performance of HCV core, to inform the design of future HCV vaccines. A way to do this would be to identify and modify or delete the domains responsible for its immunomodulating/immunosuppresive properties. For this, we characterized the molecular properties of a series of truncated HCV core variants, their performance in DNA immunization, and by linking this data attempted to delineate the molecular correlates of their immunogenicity.

A panel of HCV core variants was designed by gradual deletion of the C-terminus after aa 173, 152, 98, and 60, and of the N-terminus up to aa 36, encoded as in the HCV 1b strain 274933RU (core191v to core60v), and also HCV core aa 1–152 encoded by synthetic DNA (core152s) [58]. The highest level of expression in eukaryotic cells was achieved in the case of “viral” core191v, highly expressed in Huh7 cells, and “synthetic” core152s, well-expressed in different cell lines (Huh7, BHK, and HEK203 cells). All HCV core variants truncated either from the N-terminus, or beyond aa 152 were present at the levels up-to 100 times lower than the parental protein. MG132 and epoxomycin stabilization assays revealed that of these unstable variants, only core152 was cleaved by the proteasome. Andersson et al. have further shown that the removal of the highly hydrophobic C-terminal region from the HCV core protein relieves ER sequestration, promotes HCV core degradation via the ubiquitin-proteasome proteolytic pathway, and enhances CD8+ T cell response [85]. This, together with the observation that proteasome rapidly degrades the HCV core mutant devoid of aa 125 to 166 [86], assigns the domain responsible for the proteasomal stability/ER sequestration of HCV core to the region between aa 152 and 166. HCV core variants truncated beyond aa 152 (core60, or core98) were insensitive to proteasomal inhibitors. This can then be explained by the allocation of the domain responsible for targeting of HCV core for ubiquitination to aa 99 to 152. The latter,with the help of the data by Hope and McLauchlan [86] could be restricted to the region between aa 99 and 125.

Further, we have characterized the immunogenicity of HCV core variants in BALB/C mice after DNA immunization with electroporation (EP). Core152s DNA was superior to all other variants in the capacity to induce the specific IFN-γ, IL-2, and dual IFN-γ/Il-2 response, and anti-HCV core antibodies. DNA-immunization of C57BL/6 mice by intramuscular injections without EP confirmed the superior immunogenicity of core152s DNA over the “next-best” full-length core191v. Recipients of the core191s gene responded by a transient production of IFN-γ and IL-2, weak proliferative responses, and a low level of specific antibodies. On contrary, core152s DNA induced long-lasting specific proliferative responses of murine splenocytes, secretion of INF-γ, IL-2, and IL-4, and production of specific antibodies. Thus, in two models tested, the truncation of the C-terminal 39 amino acids of HCV core led to a profound enhancement of its immunogenic performance.

We have also assessed the effector potential of anti-HCV core immune response in vivo by co-injecting DNA immunogens together with the plasmid encoding firefly luciferase and monitoring the disappearance of the reporter activity from the sites of co-injections. In these assays, the diminishment of Luc reporter expression correlates with the development of lytic responses of CD8+ and Th1-type CD4+ T-cells specific to the immunogen [65,81,83,84]. Here, as well, the disappearance of reporter luminescence correlated with the magnitude of IFN-γ response to a peptide pool which incorporated a CTL epitope recognized in BALB/c mice (aa 41 to 50; [16,47]). Significant clearance of the reporter-expressing cells was observed only in mice immunized with core152s DNA, advancing it as the only one capable of inducing a significant effector response with a lytic potential.

Earlier, in a mouse study of the oxidative stress and oxidative stress response to a panel of genes encoding HIV-1 reverse transcriptase (RT) of varying immunogenicity, we showed that IFN-γ response induced in immunization of mice with RT DNA correlated with the capacity of encoded RT variants to induce ROS and activate the transcription of ARE-dependent phase II detoxifying enzyme genes [87]. With this in mind, we assessed HCV core variants for the capacity to induce the oxidative stress and oxidative stress response. All variants with the intact N-terminus induced an oxidative stress manifested by the production of ROS. The strongest (per unit of protein in expressing cell) effect on the production of ROS was generated by the heavily truncated core60 followed by core98. These data supported our earlier finding of a ROS-inducing domain at the N-terminus of the protein [26].

ROS trigger a strong stress response mediated by the Nuclear factor E2-related factor 2 (Nrf2) [63,88,89]. “Inactive” Nrf2 is retained in the cytoplasm by Keap1 protein. ROS trigger Nrf2 phosphorylation, release from Keap1 and translocation into the nucleus, followed by the antioxidant response element (ARE) dependent induction of expression of Phase II detoxifying enzymes [90]. Here, as well, generation of ROS was followed by a three to six-fold up-regulation of the expression of Phase II detoxifying enzymes heme oxygenase 1 (HO-1) and NAD(P)H: quinone oxidoreductase (Nqo-1) on the transcriptional and translational levels. Interestingly, while reducing the levels of ROS production, truncations within the C-terminal 39 amino acids had little effect on the parameters of the oxidative stress response normalized to expression of respective proteins, with concordant results in the transcription and translation assays. Cumulatively these data indicate that the expression Nqo-I and HO-1 was induced by a domain within aa 1–60 of HCV core, which falls in line with its ability to up-regulate the expression of ROS generating enzymes, NADPH oxidase 1 and 4 (Nox1 and Nox4) [26]. Previously, we found that two HCV core domains aa 1–36 and 37–191 can activate the Nrf2/ARE pathway, albeit with different efficacy and via different mechanisms [89]. Both HCV core aa 1–36 and aa 37–191 activated Nrf2, aa 1–36 through phosphoinositide-3-kinase (PI3K and casein kinase 2 (CK2) by a ROS-independent, and aa 37–191 by a classical protein kinase C (PKC)- and ROS-dependent mechanism.

In the next step, we have correlated the immunogenic performance of HCV core variants with their capacity to induce the oxidative stress and oxidative stress response. The IFN-γ response of mice DNA-immunized with HCV core (as earlier of the recipients of HIV RT DNA [87]) correlated with the ability of gene immunogens to generate ROS and induce the expression of Nqo-1 and HO-1. At the same time, the response was affected by high relative levels of transcription/translation of Nqo-1 and HO-1 (per unit of HCV core protein expressed in a cell), i.e., depended on a balance between the stress and the stress response. This interdependence could explain weak immunogenicity of heavily-truncated HCV core variants, which expressed at a low level, induced high levels of ROS, and a strong oxidative stress response. No significant correlations were observed between cellular immunogenicity (in terms of cytokine production) and protein stability, or the route of protein degradation,although we saw a tendency for correlation between the IFN-γ response and susceptibility of HCV core variants to proteasomal degradation reflected by the degree of stabilization of HCV core variants by the proteasomal inhibitor epoxomycin.

The role of Phase II detoxifying enzymes in the development of immune response is not fully appreciated. HO-1 represses inflammation by removing the pro-inflammatory heme molecule protecting from oxidant injury [90,91], and can indirectly modulate the immune response [92,93]. Its reaction products appear to block the innate and modify the adaptive immune responses by affecting the activation, differentiation, maturation, and polarization of numerous immune cells, including endothelial cells, monocytes/macrophages, dendritic cells, T lymphocytes, mast cells, and platelets [91]. HO-1 is constitutively expressed in a subpopulation of T regulatory cells (CD4+ CD25+), with levels increasing after their stimulation [94,95]. In liver allografts, high HO-1 levels correlate with the predominance of Th2-cytokines IL-4 and IL-10, and low HO-1 levels of Th1-cytokines IFN-γ and IL-2 [96,97]. Deficiency in HO-1 predisposes to a dramatic increase in pro-inflammatory cytokines following macrophage stimulation with the predominant production of Th1-type cytokines as IFN-γ and TNF-α [97]. Much less is known about Nqo-1. It is a cytosolic enzyme that catalyzes the metabolic reduction of quinones and derivatives. Studies in the animal models demonstrated its potential role in the protection against the cardiovascular injury and related conditions [98], and control of the immune response versus autoimmunity [99]. Hereby, on the example of BALB/c mice DNA-immunized with HCV core, we found that the induction of HO-1 and Nqo-1 exerts a concert effect on the immune system manifested by the suppression of production of IFN-γ. Their upregulation may be also involved in the Th2-tilting of immune response reflected by secretion of IL-4 and production of specific antibodies.

As we have earlier shown, ROS production in response to HCV core involves Nox1 and Nox4, and also cytochrome P450 2E1 (CYP2E1) [26]. The CYP2E1-inducing domain resides within aa 38–191 of HCV core protein [26]. Hereby, we found that truncations within the C-terminal 39 amino acids result in the loss of ROS-inducing protein domain, reflected by reduction in the normalized levels of ROS. This together with our earlier data [26] indicate that CYP2E1 inducing domain may localize within aa 153–191 of HCV core protein. Cytochrome 2E1 is a key enzyme of the cytochrome P450 family in the metabolic activation of a variety of xenobiotics and carcinogens [100], ethanol-induced oxidative stress and alcohol liver injury [101]. It has a high NADPH oxidase/ROS generating activity and is the most efficient P450 enzyme in the initiation of the NADPH-dependent lipid peroxidation and oxidative DNA damage, contributing significantly to the tissue injury in the pathological, as well as experimental settings [100,102,103,104]. Cells overexpressing CYP21E release ROS and diffusible mediators such as the lipid peroxidation products which can affect the neighboring cells. These mediators elicit an inflammatory response in both expressing and “innocent” neighboring cells [101], including cells of the immune system. Indeed, in MRL^+/+^ mice, substrate-induced activation of CYP2E1 promoted the expansion of the population of Th1-type CD4 T cells, and accelerated the development of an autoimmune response [105,106]. CYP2E inhibition reversed these alterations [105,106]. In line with this, we observed Th1-skewing of the immune response in mice DNA-immunized with the full and nearly full length HCV core (core173v and core191v), and Th2-skewing in mice DNA-immunized with HCV core devoid of the C-terminus. CYP1E2 activity may explain the inhibitory effect of HCV core on the immune response against the co-delivered DNA immunogens [16,107]. Interestingly, CYP1E2 is also involved in the inhibition of the proteasomal processing [108]. We could then expect that the removal of the C-terminal HCV core domain inducing CYP2E would restore the immune response.

The C-terminal domain of HCV core protein is involved in the immune regulation in different ways. One is through NF-κB protein, a central mediator of the immune response [35]. NF-κB modulating activity is lost with the loss of HCV core C-terminus [109]. The latter is involved in the down-regulation of expression from the NF-κB/Rel regulated promoter of the IL-2 gene; its deletion abrogates the suppression [110]. Another crucial regulator of the immune system is the cyclin-dependent kinase inhibitor p21/Cip1/WAF1. It modulates the macrophage activation, production of inflammatory cytokines [111,112], and development of the adaptive immune response [113,114]. HCV core interferes with the p21 regulation by reducing its expression [115,116]. Also in this case, the negative effects of HCV core on p21/Waf1 activity are abrogated by the deletion of HCV core C-terminus [117].

Interference of one and the same domain in several cellular cascades indicates that HCV core acts at a common stage(s) (possibly regulated by the same affected transcription factor). In this context, it is crucial to mention the association of HCV core with the mitochondria (mt) mediated by the C-terminal domain of HCV core protein [75,118]. HCV core binding to and within the mitochondria profoundly alters their functions in the metabolism, redox balance, ROS scavenging and apoptosis. Domain responsible for mt targeting is localized at aa 139 to 158, but even a shorter C-terminal 10-amino-acid motif seems to be sufficient [75]. The interaction of HCV core with mt increases Ca^2+^ entry and subsequently elevates generation of superoxide anions by mitochondrial electron transport complex I [119]. This results in the mitochondrial GSH and mt depolarization with drastic increases in ROS production, augmented by the simultaneous ER stress [119,120]. In its turn, ROS flux leads to the opening of the mitochondrial permeability transition pores, and the release of cytochrome C or Ca^2+^ resulting in apoptosis [118]. This has been shown in isolated mitochondria, cells expressing HCV core, cells harboring the full-length replicon, and in the liver mitochondria derived from the transgenic mice [119,121]. Mt injury and resulting flux of ROS may, in fact, be a common trigger of the deregulation of NF-kB, p21/WAF, as well as STAT signaling [88]. It seems logical to exclude a domain with such deleterious properties from the prototype HCV vaccines as potentially harmful to the host.

## 5. Conclusions

In conclusion, our study demonstrated that cellular immunogenicity of HCV core is shaped by its ability to induce ROS and oxidative stress response. In two mouse models tested, the truncation of the C-terminal 39 amino acids of HCV core led to profound changes in its immunogenic profile manifested by (i) the increased magnitude of cellular responses; (ii) enhanced B-cell responses; (iii) multifunctionality (in vitro response multiple cytokines); and (iv) increased response longevity. The immunogenic performance of HCV core genes did not depend on the route (ID or IM) and mode of DNA delivery (with or without electroporation). All approaches proved the superior immunogenicity of the C-terminally truncated HCV core encoded by the synthetic gene, which we have linked to its capacity for a balanced induction of oxidative stress and oxidative stress response. Our analysis of the molecular properties of HCV core in relation to its immunogenicity in mice revealed that domain involved in the immune suppression localizes to the C-terminal aa 153–191 of the protein. This may inform the design of new HCV vaccine candidates with improved immunogenic performance.

## Figures and Tables

**Table 1:** Average IFN-γ production by splenocytes of HCV core DNA immunized mice after in vitro stimulation with HCV core-derived peptides correlate to the ability of respective core gene to induce ROS production and oxidative stress response. R values reflecting significant correlations are given in red (*p* < 0.05; Spearman ranking test).

Parameter	Correlates				
	Total IFN-γ Production to All Peptides	IFN-γ Core 1-152	IFN-γ Core Peptide Pool 1 ^1^	IFN-γ Core Peptide Pool 2 ^2^	IFN-γ Core Peptide Pool 3 ^3^
ROS	0.857143	0.882919	0.637748	0.600000	0.637748
Nqo1	0.750000	0.774806	0.637748	0.600000	0.637748
HO-1	0.571429	0.540562	0.985611	0.942857	0.811679
ROS normalized to vector	0.535714	0.504525	0.927634	0.885714	0.753702
Nqo1 normalized to vector	0.821429	0.792825	0.985611	0.942857	0.811679
HO-1 normalized to vector	0.785714	0.756787	0.927634	0.885714	0.753702
ROS normalized to effect of core152v	−0.750000	−0.774806	−0.637748	−0.600000	−0.637748
Nqo1 normalized to effect of core152v	−0.857143	−0.882919	−0.811679	−0.771429	−0.637748
HO-1 normalized to effect of core152v	0.857143	0.882919	0.637748	0.600000	0.637748

^1^ Pool 1—aa 1–56 (1–20, 13–33, 34–42, 34–56); ^2^ Pool 2—aa 63–126 (63–80, 76–90, 106–126). ^3^ Pool 3—aa 129–177 (129–145, 141–160, 155–177).

## Supplementary Materials

The following are available online at https://www.mdpi.com/2073-4409/8/3/208/s1, Figure S1. Detection of HCV core variants in Huh7 and BHK-21 cells by immunofluorescence microscopy; Figure S2. Expression of HCV cores gene variants in BHK-21 cells characterized by confocal microscopy; Figure S3. The stability of HCV core protein variants in BHK-21 cells assessed by the cycloheximide-chase; Figure S4. Cellular immune response of BALB/C mice to DNA-immunization with HCV core aa 1–191; Figure S5. Antibody response of BALB/c (A) and C57Bl/6 (B) mice to DNA-immunization with HCV core gene variants.
