# Supplementary material for: The Immunogenicity in Mice of HCV Core Delivered as DNA Is Modulated by Its Capacity to Induce Oxidative Stress and Oxidative Stress Response
Source: Cells. 2019 Feb 28;8(3):208. doi: 10.3390/cells8030208 (PMC6468923; doi:10.3390/cells8030208)
Supplement: Supplementary file 1 [file cells-08-00208-s001.pdf]

Supplement to

The Immunogenicity in Mice of HCV Core Delivered as DNA Is Modulated by Its Capacity to Induce Oxidative Stress and Oxidative Stress Response

Juris Jansons [1,2,3†], Irina Sominskaya [2†], Natalia Petrakova [4], Elizaveta S. Starodubova [4,5], Olga A. Smirnova [5], Ekaterina Alekseeva [1,2], Ruta Bruvere [2], Olesja Eliseeva [4], Dace Skrastina [1,2], Elena Kashuba [3,6], Marija Mihailova [2], Sergey N. Kochetkov [5], Alexander V. Ivanov [5] and Maria G. Isaguliants [1,3,4,7*]

[1] Department of Pathology, Riga Stradins University, LV-1007 Riga, Latvia; juris.jansons@rsu.lv (J.J.)

[2] Latvian Biomedical Research and Study Centre, Ratsupites iela 1, Riga LV1067, Latvia; Irina@biomed.lu.lv (I. S.); kate@ biomed.lu.lv (E. A.); diana.legzdina@inbox.lv (D. L.); Bruvere@biomed.lu.lv (R.B.); mary@biomed.lu.lv (M.M.) ; kate@biomed.lu.lv (E.A.); daceskr@biomed.lu.lv (D.S.)

[3] Department of Microbiology, Tumor and Cell Biology, Karolinska Institutet , SE-171 77 Stockholm Sweden; estarodubova@gmail.com (E. S.); maria.issagouliantis@ki.se (M.I.)

[4] N.F. Gamaleya Research Center of Epidemiology and Microbiology, Ministry of Health of the Russian Federation, Gamaleya str. 18, Moscow 123098, Russia; nvpetrakova@hotmail.com (N. P.); olesenka80@mail.ru (O.E.)

[5] Engelhardt Institute of Molecular Biology, Academy of Sciences of the Russian Federation, Vavilova str. 32, Moscow 119991, Russia; o.smirnova.imb@gmail.com (O. S.); estarodubova@gmail.com (E. S.); kochet@eimb.ru (S. K.); aivanov@yandex.ru (A. I.)

[6] RE Kavetsky Institite of Experimental Pathology, Oncology and Radiobiology, the National Academy of Sciences of Ukraine, Vasylkivska Str 45, 03022 Kyiv, Ukraine; elena.kashuba@ki.se (E.K.)

[7] MP Chumakov Center for Research and Development of Immune and Biological Preparations of RAS, 108819 Moscow, Russia: maria.issagouliantis@rsu.lv (M.I.)

† These authors contributed equally to this work.

* Correspondence: maria.issagouliantis@rsu.lv; Tel.: +37125244801

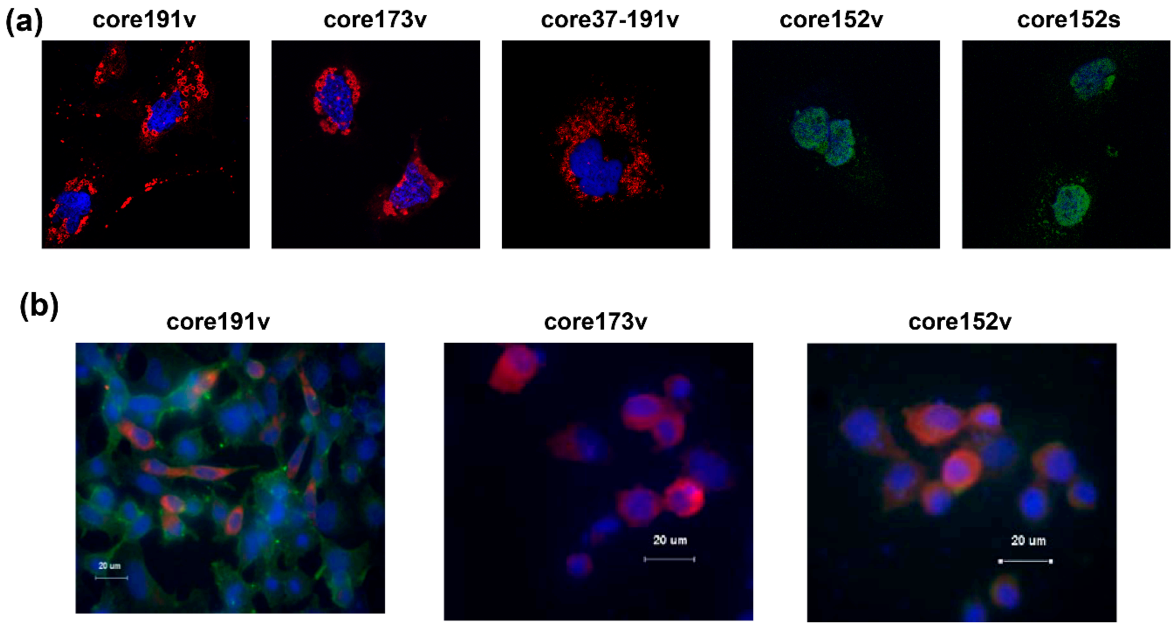

Figure S1. Detection of HCV core variants in Huh7 and BHK-21 cells by immunofluorescence microscopy. (**a**) Core gene variant transfected into a cell line is depicted on top of each slide. In Huh7 cells, HCV core protein variants were detected by the immunofluorescent staining using HCV core-specific rabbit polyclonal as primary [1] and TRITC-conjugated (core191v, core173v, and core37-191v; red) or FITC-conjugated anti-rabbit secondary antibodies (core152v and core152s; green); and (**b**) in BHK-21 cells, HCV core protein variants were detected by the immunofluorescent staining using HCV core-specific rabbit polyclonal as primary [1] and TRITC-conjugated secondary antibodies; WGA 488 was used to visualize the cell membrane (green). The nucleus was visualized by DAPI staining (blue). Fluorescent images were visualized on Leica DM 6000 B microscope (Leica, Wetzlar, Germany) and recorded with a Leica DFC 480 camera.

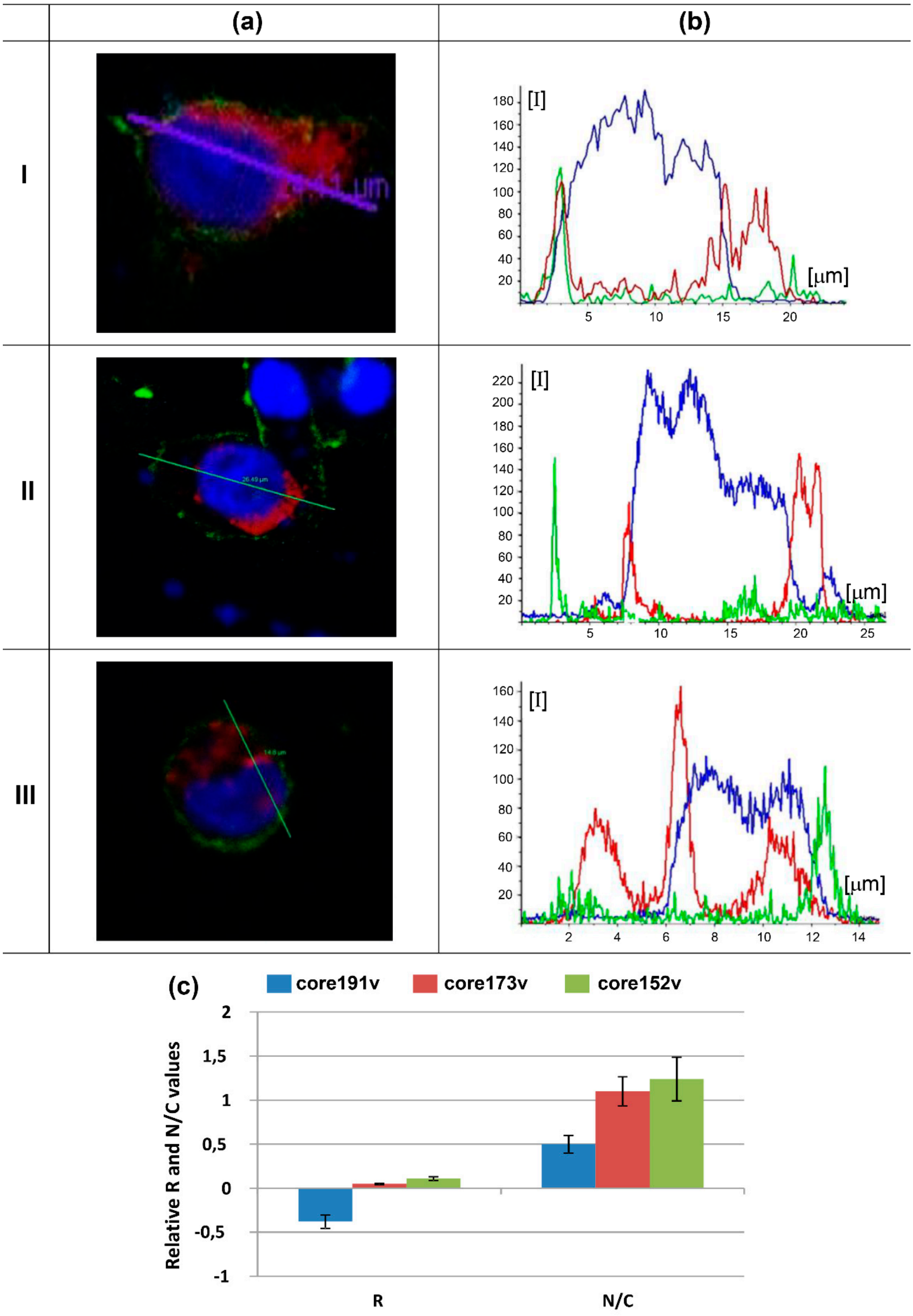

Figure S2. Expression of HCV cores gene variants in BHK-21 cells characterized by the confocal microscopy. BHK-21 cells were transfected with core191v (I), core173v (II), or core152v (III) DNA, and analyzed 24 h post transfection for the expression of HCV core variants by staining first with core-specific rabbit polyclonal antibody [1] and then with TRITC-conjugated anti-rabbit secondary antibodies (red). Nuclei were visualized by staining with DAPI (blue), and cell membranes, with WGA 488 (green). (**a**) Representative images of cell slices obtained by laser scanning confocal microscopy (laser microscope Leica TCS SP2 SE); (**b**) quantification of the images in panel (**a**) reflecting the fluorescence intensity of each color in a single cell along the cutting

line; and (**c**) the ratio between the nuclear and cytoplasmic portions (N/C ration) and coefficient R = (N – C) / (N + C) calculated based on the image quantifications as described by Leclerc et al. [2]. Quantifications done on other image selections differed by less than 20%. N/C and R values for core191 were statistically different from those for core173 and core152 ($p < 0.05$; Tukey–Kramer test).

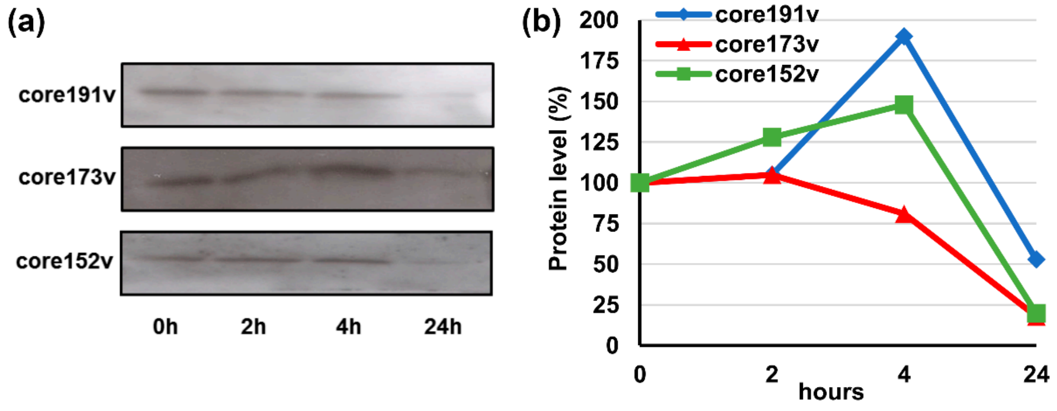

Figure S3. The stability of HCV core protein variants in BHK-21 cells assessed by the cycloheximide-chase. (**a**) Expression of HCV core protein variants directed by plasmids carrying corresponding genes in BHK-21 cells; (**b**) quantification of expression using ImageJ software At 24 h post-transfection, cycloheximide was added to cells cultures to a final concentration of 100 µM, cells were sampled and lysed immediately after, or 2, 4, or 24 h post cycloheximide addition. Western blotting was done with the polyclonal anti-core rabbit sera and HRP-labeled goat anti-rabbit antibodies.

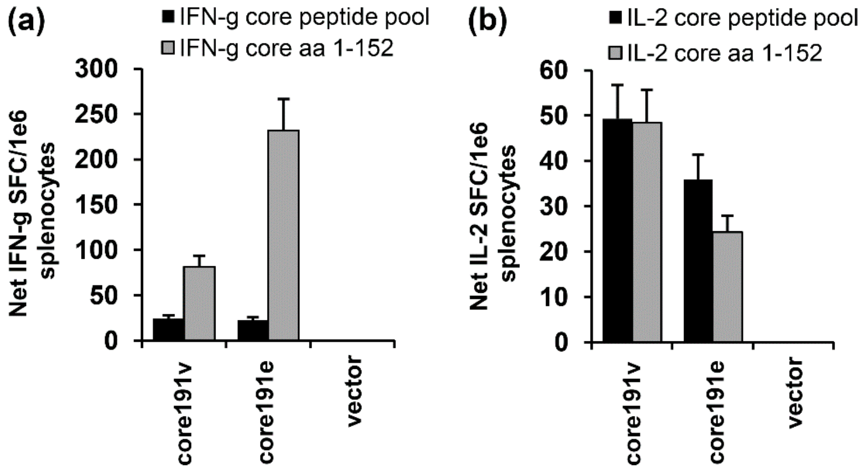

Figure S4. Cellular immune response of BALB/C mice to DNA-immunization with HCV core aa 1–191. Core191 was encoded by viral sequenced under control of immediate early cytomegalovirus promoter IE CMV (core191v) (**a**) or human elongation factor 1-alpha promoter (core191e) (**b**). Respective plasmids were delivered by intradermal injections followed by electroporation. Graphs represent cytokine secretion of mouse splenocytes responding to in vitro stimulation with core-derived peptide pool and recombinant core152 by production of IFN-γ (**a**) or IL-2 (**b**). All assays were conducted in duplicate. Results represent the average values for all mice in the group ± SD. No difference between the groups was registered in response to stimulation with the mitogen ConA ($p > 0.05$; data not shown).

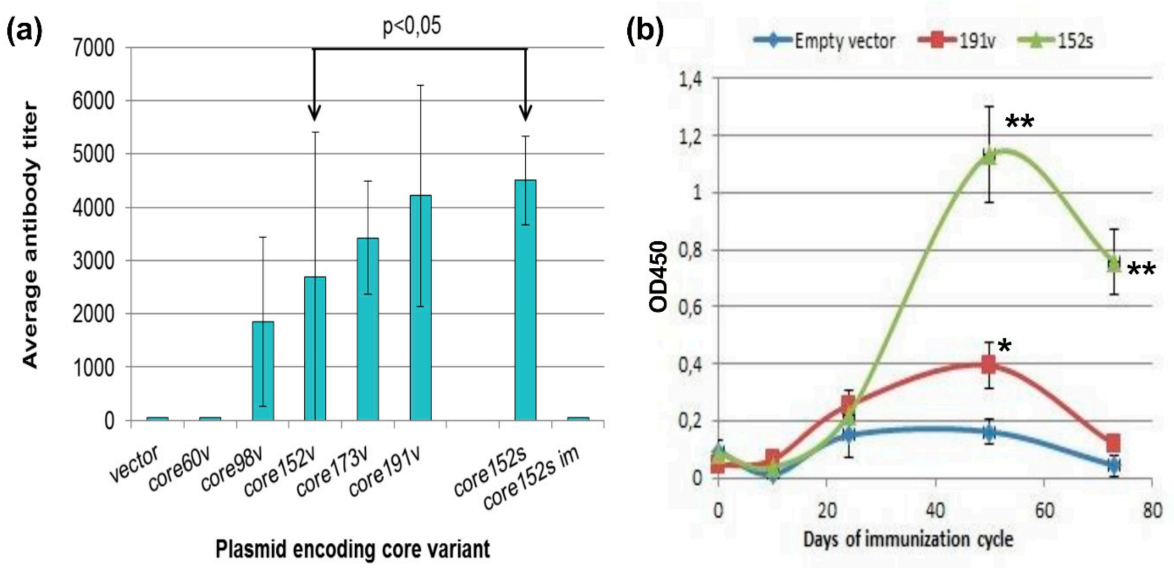

Figure S5. Antibody response of BALB/c (**a**) and C57Bl/6 (**b**) mice to DNA-immunization with HCV core gene variants. (**a**) Average antibody titer ± STDEV of anti-HCV core antibodies in BALB/c mice (n = 3–6 per group) DNA-immunized with core variants encoded by virus-derived sequence core60v, core98v, core152v, core173v, core191v, and synthetic DNA core152s introduced intradermally with electroporation, or intramuscularly with Turbofect (core152s im); and (**b**) dynamics of development of anti-core antibody response (OD450 ± STDEV) in C57BL/6 mice (n = 20 per group) immunized by intramuscular injections of core191v, or core152s DNA, or empty vector at month 1, 2, and 3, and assessed before immunization, 1.5–2 weeks after prime, before 1st boost, and two weeks post 1st and 2nd boosts (n = 4–20 per time point). Sera was diluted 1:400. Serum reactivity was evaluated by indirect ELISA on plates coated with recombinant core152. * $p < 0.05$ in core191v DNA immunized mice compared to vector mice; ** $p < 0.01$ in core152s DNA immunized mice compare to control mice.

Additional references

1. Isaguliants, M.G.; Petrakova, N.V.; Kashuba, E.V.; Suzdaltzeva, Y.G.; Belikov, S.V.; Mokhonov, V.V.; Prilipov, A.G.; Matskova, L.; Smirnova, I.S.; Jolivet-Reynaud, C., *et al.* Immunization with hepatitis c virus core gene triggers potent t-cell response, but affects cd4+ t-cells. *Vaccine* **2004**, *22*, 1656-1665, doi: 10.1016/j.vaccine.2003.09.047.
2. Leclerc, P.; Jibard, N.; Meng, X.; Schweizer-Groyer, G.; Fortin, D.; Rajkowski, K.; Kang, K.; Catelli, M.G.; Baulieu, E.E.; Cadepond, F. Quantification of the nucleocytoplasmic distribution of wild type and modified proteins using confocal microscopy: Interaction between 90-kda heat shock protein (hsp90 alpha) and glucocorticosteroid receptor (gr). *Experimental cell research* **1998**, *242*, 255-264, doi:10.1006/excr.1998.4117.
